# Differential effects of caffeine, acute aerobic exercise, and placebo on mental fatigue

**DOI:** 10.1371/journal.pone.0348279

**Published:** 2026-04-30

**Authors:** Ali Shirzad, Nathan Frewen, Anisa Morava, Nader Elshawish, Harry Prapavessis

**Affiliations:** 1 Department of Kinesiology, Faculty of Health Sciences, Western University, London, Ontario, Canada; 2 Schulich School of Medicine and Dentistry, Western University, London, Ontario, Canada; 3 Temerty Faculty of Medicine, University of Toronto, Toronto, Ontario, Canada; Poznan University of Physical Education, POLAND

## Abstract

Mental fatigue negatively affects the productivity and health of the general population. Mental fatigue manifests following sustained mental activity and is defined by subjective feelings of inhibition and objective decrements in mental performance. Caffeine is one of the most common stimulants used to mitigate mental fatigue. However, regular caffeine consumption may be associated with several side effects and potential dependence. The efficacy of acute aerobic exercise as an alternative mental fatigue intervention remains ambiguous. Thus, the present study utilized a randomized crossover design to compare the efficacy of the three treatments in mitigating mental fatigue in 26 adult caffeine consumers. The three treatments included: 1) 20 minutes of moderate-intensity cycling, 2) consumption of 2.5 mg/kg of caffeine, and 3) consumption of placebo (0.67 g corn starch). Treatments were administered prior to a mental fatigue-induction task (30-minute Stroop task). Participants and the experimenter were both blinded to the administration of caffeine or placebo. Objective (Psychomotor Vigilance Task) and subjective (visual analog scales) mental fatigue measures were taken prior to and following the mental fatigue-induction task. We hypothesized that in the placebo condition, participants would demonstrate greater mental fatigue following a 30-minute Stroop task relative to baseline. Further, mental fatigue induction will be mitigated in the acute exercise and caffeine consumption conditions to a similar degree. Participants reported significantly greater mental fatigue following a 30-minute Stroop task (p < 0.001, d = −0.735). Comparison of the placebo and caffeine consumption did not exhibit a reliable difference (all p’s > 0.05). Contrary to our hypothesis, mental fatigue was not mitigated following acute exercise (p = 0.499). Further investigations are warranted to explore the potential of varying exercise intensities and durations in mitigating mental fatigue.

## Introduction

Fatigue is a multidimensional concept, commonly understood as feelings of tiredness and difficulty in initiating or sustaining voluntary activity following prolonged mental or physical activity [1]. Fatigue is often characterized by its subjective experience and effects on task performance [2]. Specifically, fatigue that manifests as cognitive impairment is known as mental fatigue [3]. Accruing mental fatigue through sustained mental activity can be specifically defined as “a gradual and cumulative process … associated with a disinclination for any effort, a general sensation of weariness, feelings of inhibition and impaired mental performance, reduced efficiency and alertness” [4]. Indeed, mental fatigue has been shown to disrupt several cognitive processes including attention, planning, decision-making, inhibitory control, and memory [5–8]. The feeling of mental fatigue (i.e., subjective fatigue) may fluctuate independent of task performance (i.e., objective fatigue); thus, it is important to measure both constructs [9,10]. Approximately 50% of U.S. adults report experiencing moderate-to-severe fatigue throughout the course of a week [11]. Though fatigue is widely accepted as a part of daily life, it has been associated with several unwanted outcomes. For example, fatigue in U.S. workers has been estimated to account for “an excess of $101.0 billion” in lost productive time annually [12]. Further, one of the most prevalent symptoms affecting individuals seeking primary care is fatigue, which is thought to “account for 10 million office visits a year in the [U.S.]” [13]. Thus, interventions to mitigate fatigue are necessary to support the overall productivity and health of the general population.

Caffeine (1,3,7-trimethylxanthine) consumption reliably mitigates mental fatigue [14–17]. Caffeine exerts this effect on the central nervous system through antagonism of central adenosine receptors [18]. Approximately 89% of adults in the U.S. consume caffeine regularly [19]. Moreover, daily caffeine consumption among adults has been estimated to be between 1.7–2.9 mg/kg [20]. Past research has also demonstrated the beneficial effects of caffeine consumption on alertness, mood, attention, and cognitive task performance [21–24]. Although the benefits of caffeine consumption are well-documented, these may be concurrent with several undesirable side effects. Acute side effects of caffeine consumption include anxiety, muscle tremors, and gastrointestinal distress [25–27]. Further, habitual caffeine consumption has been associated with withdrawal symptoms including fatigue, headache, nausea, and depressed mood, which may manifest after 12–24 hours of caffeine deprivation [28–30]. Several authors have suggested that caffeine dependence can develop after habitual, high-dosage use [31,32]. However, the formal inclusion of caffeine substance use order in diagnostic manuals such as the DSM is an area of active contention [33,34].

Altogether, a large body of evidence suggests caffeine usage, although effective in mitigating mental fatigue, may result in side effects and potential dependence. Thus, it is important to explore alternative methods for mitigating mental fatigue. Past research has amply demonstrated that a single bout of aerobic exercise (i.e., acute aerobic exercise) can enhance cognitive function [35–38]. Additionally, work by Morava and colleagues [39] suggests that acute moderate-intensity aerobic exercise may reduce caffeine withdrawal symptoms. Therefore, acute aerobic exercise may be a promising alternative to caffeine consumption; however, its effects on mental fatigue are unclear. To our knowledge, no prior studies have directly compared caffeine and aerobic exercise as fatigue-mitigation strategies. Thus, the present exploratory study was designed to evaluate their individual effects before exploring potential combined interventions in future work.

Two mechanisms that potentially underlie acute exercise benefits to cognitive function are: 1) increased cerebral blood flow (CBF), and 2) the modulation of neurotransmitter concentrations [40–42]. Increases in CBF following exercise have been well-documented and are thought to arise from neurovascular coupling, increased CO_2_ production, and increased systolic blood pressure (BP) [43,44]. Furthermore, production of several neurotransmitters has been suggested to increase in response to acute aerobic exercise, including dopamine, noradrenaline, adrenaline, acetylcholine, glutamate, and gamma-aminobutyric acid [42,45–48]. Broadly, in line with Yerkes & Dodson’s [49] arousal-performance theory, the production of these neurotransmitters, at specific exercise intensities, may help increase cognitive arousal and thus enhance cognitive performance.

Several past studies have suggested that shorter bouts of aerobic exercise may mitigate mental fatigue. Jacquet et al. [50] and Oberste et al. [51] found that completing a 15–to–30-minute bout of moderate-intensity exercise following mental fatigue induction resulted in better recovery of motor control and cognitive flexibility, respectively. Later, Ahn et al. [52] found that 20 minutes of passive (i.e., motor-driven movement) and light-intensity active exercise completed during mental fatigue induction mitigated a mental fatigue-induced inhibitory control deficit (measured via anti-saccade task).

Contrastingly, there has been some evidence suggesting that lengthy (≥40 minutes), moderate-to-high-intensity bouts of exercise may induce mental fatigue. In Moore et al.’s [53] randomized controlled trial, participants (n = 30; 15 female) completed a 40-minute memory vigilance test and perceptual discrimination test following either 60 minutes of moderate-intensity cycling, or seated rest (control). Results showed the cycling group had worse performance on both the vigilance and perceptual-discrimination tasks compared to the control group, post-intervention. Similarly, Alder et al. [54] reported that participants (n = 16) who completed a 40-minute bout of high-intensity interval training experienced a decrement in performance on a subsequent soccer-specific video anticipation assessment. Further, the decrement in performance was comparable to participants who, instead of exercise, completed a 30-minute mentally fatiguing Stroop task.

Until recently, no past investigations had examined the effect of acute aerobic exercise when completed *prior* to mental fatigue induction, which has important practical implications given its utility as a fatigue mitigation strategy (e.g., a student aiming to sustain attention during a lengthy lecture). Jeyarajan et al [55]. investigated the effect of a 20-minute bout of moderate-intensity exercise on subsequent inhibitory control and mental fatigue resilience. In their procedure, participants completed 20 minutes of moderate-intensity exercise, or passive cycling, or seated control, a 13-minute saccade task to evaluate inhibitory control, then finally a 20-minute Psychomotor Vigilance Task (PVT) to induce and evaluate mental fatigue. Participants demonstrated improvements to their inhibitory control in all three conditions, but no reliable improvement to their mental fatigue resilience. Furthermore, differences in inhibitory control and mental fatigue were not related to alterations in CBF. Several limitations of this work warrant further investigation: 1) mental fatigue was not induced immediately after exercise; 2) PVT was not completed at baseline (i.e., prior to exercise or saccade task); 3) PVT served as an induction and measurement of mental fatigue; and 4) exercise was not compared to an intervention that reliably mitigates mental fatigue.

**Fig 1:**
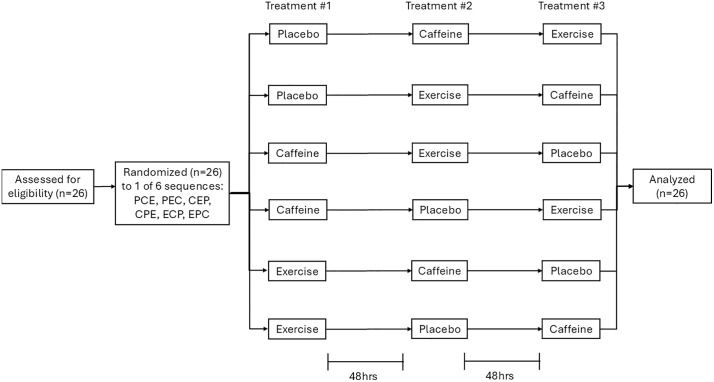
Study Design.

**Fig 2:**
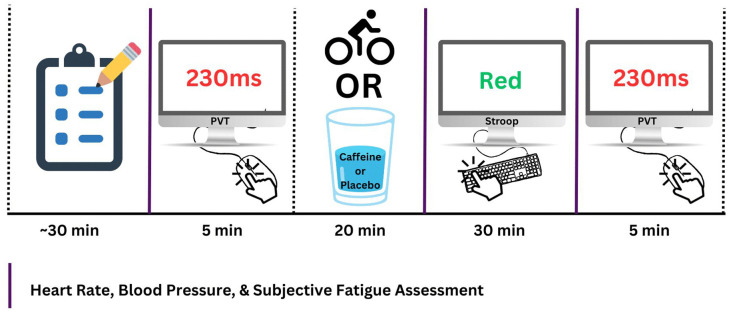
Procedural Schematic.

Thus, the objective of this investigation was to compare the effects of three treatments on objective and subjective assessments of mental fatigue when administered immediately prior to mental fatigue induction: 1) a 20-minute, moderate-intensity bout of cycling; 2) consumption of 2.5 mg/kg caffeine; and 3) placebo. To compare these three treatments, we utilized a crossover design in which participant and experimenter were both blinded to the administration of caffeine and placebo. A 30-minute Stroop task was used for the purposes of fatigue induction. Objective mental fatigue was operationalized as participants’ performance on the psychomotor vigilance task (PVT), and subjective mental fatigue was operationalized as participants self-reported score on visual analog fatigue and energy scales. We hypothesized that 1) in the placebo condition, participants will demonstrate greater mental fatigue following a 30-minute Stroop task relative to baseline (i.e., mental fatigue induction), and 2) mental fatigue induction will be mitigated in the acute exercise and caffeine consumption conditions, to a similar degree. Fig 1 depicts a flow chart of the study design. Furthermore, Fig 2 provides a schematic of participants’ in-lab sessions.

## Materials and methods

### Sample power calculation

The sample size was determined *a priori* using the G*Power software [56]. The following parameters were used in the sample size calculation: power = 0.8, alpha = 0.05, and an effect size of η_p_^2^ = 0.4. Rubio-Morales et al. [57] measured mental fatigue induction following a 30-minute Stroop task using a 3-minute PVT reaction time. The effect size of the post-fatigue induction increase in RT was approximately 0.594. Due to the novel acute exercise mental fatigue intervention, a more conservative effect size estimate of 0.4 was chosen. The sample size calculation yielded a target of 21 participants.

### Participants

Twenty-six undergraduate and graduate students from the Western University community were recruited to participate in this study (see Table 1 for participant characteristics). The inclusion criteria were as follows: 1) aged 18–30 years, 2) consume 150–500 mg of caffeine daily, and 3) be able to read and write in English. The exclusion criteria were as follows: 1) tobacco, marijuana, or other recreational drug use, 2) prescription medication use for chronic health conditions (e.g., depression, anxiety, cardiovascular conditions, endocrine conditions), 3) diagnosed with a colour vision deficiency, 4) contraindications to exercise as identified by the Physical Activity Readiness Questionnaire for Everyone (PAR-Q+), 5) pregnancy, or breastfeeding.

**Table 1:** Participant Characteristics.

Sample size	n = 26
Age, years, mean (SD)	23.3 (3.0)
Sex, female, *n* (%)	14 (53.8)
Weight, kilograms, mean (SD)	73.7 (16.0)
Education, years, mean (SD)	17.0 (2.4)
Daily Caffeine, milligrams, mean (SD)	331.9 (122.1)
Sleep, hours, mean (SD)	7.9 (1.0)
GLTEQ, mean (SD)	57.1 (23.8)
CWSQ, mean (SD)	19.4 (7.6)
Trait mental fatigue, mean (SD)	4.1 (1.7)
Trait mental energy, mean (SD)	6.7 (1.5)
Trait physical fatigue, mean (SD)	3.8 (1.5)
Trait physical energy, mean (SD)	6.8 (1.6)

*Note. Godin Leisure-time Exercise Questionnaire (GLTEQ), Caffeine Withdrawal Symptom Questionnaire (CWSQ).*

Only participants who reported caffeine consumption between 150–500 mg daily were selected to target average habitual caffeine users (i.e., those who have likely experienced beneficial effects from caffeine consumption). This cutoff is in line with the average daily caffeine intake reported in university-aged populations worldwide [58–60]

The upper cutoff of 500 mg/day was selected to preclude outliers with relatively high caffeine consumption who may be experiencing health risks and significant withdrawal with caffeine cessation [61–63].

Prior to data collection, this study obtained ethical approval from Western University’s Health Sciences Research Ethics Board (#121715). This study also adhered to the most recent iteration of the Declaration of Helsinki, with the exception that participants were not registered in a database [64]. The recruitment and data collection for this study was conducted between May 23^rd^, 2023, and August 2^nd^, 2024, at Western University in London, Ontario, Canada.

### Exercise treatment

The exercise intervention consisted of 20 minutes of active cycling at 70 rpm using a Monark 839e cycle ergometer (Monark, Vansbro, SE). The 20-minute bout consisted of a 2.5-minute warm-up, 15 minutes of cycling at a moderate intensity, and a 2.5-minute cool-down. Moderate-intensity exercise was defined as 50% of heart rate reserve (HRR; [65]). HRR target was calculated using the formula HRR = ((220 – age – HR_rest_) x 0.5) + HR_rest_ [66]). HR was monitored throughout the cycling bout using a wearable heart rate monitor (Polar H10 Wearlink + Coded Transmitter, Polar Electro Inc., Lake Success, NY, USA). A researcher monitored and adjusted the resistance of the cycle ergometer to ensure the participants’ HR remained within 5% of the 50% HRR target.

### Caffeine treatment

The caffeine consumption treatment consisted of participants orally ingesting a powdered caffeine solution. Each participant ingested 2.5 mg/kg (body weight) of powdered caffeine (Sigma–Aldrich Foundation, St Louis, MO) dissolved in 200 mL of water [67,68]. Participants then had 20 minutes of seated rest to permit caffeine absorption and maintain consistency with the duration of the exercise [39]. Caffeine consumption between 0.5–4 mg/kg of body mass has been shown to reliably improve alertness, vigilance, and attention [69–71]. A dose of 2.5 mg/kg falls within the tested range and has been shown to produce the greatest task-induced BOLD response compared to those observed at lower or higher doses [67].

### Placebo treatment

Similar to the caffeine treatment, the placebo consumption treatment consisted of participants orally ingesting a colour-matched mixture of 200 mL of water and approximately 0.67 g corn starch (i.e., a quarter teaspoon), followed by 20 minutes of seated rest [72].

### Mental fatigue induction

A 30-minute Stroop task was completed following treatment to induce mental fatigue [73,74]. Several past studies have demonstrated that tasks requiring response inhibition, such as the Stroop task, are effective at inducing mental fatigue [1,75–78]. Moreover, the mental fatigue induction from the Stroop task may be greater than other popular fatigue induction tasks, such as the PVT [74]. The Stroop task was completed using Inquisit software (version 4.0.8.0; Millisecond Software, 2008). The parameters of the task have been previously described by Kowal et al. [79] and Toth et al. [80]. Reaction time (RT; time in milliseconds between stimulus presentation and a participant’s response) and accuracy (proportion of correct responses) were recorded for each trial.

### Measures

#### Demographics.

Participants were asked to report age in years, sex, education (e.g., first-year undergraduate student), hours of sleep prior to lab arrival (bedtime and wake-up time), and body weight (kg). The Godin Leisure-Time Exercise Questionnaire (GLTEQ) [81] was given to participants at their first experimental session to gauge participants’ weekly physical activity levels. As a questionnaire, the GLTEQ requires minimal technical expertise and equipment to administer. The construct validity and reliability of the GLTEQ have been supported by several past studies [82–84]. Additionally, past research has suggested that GLTEQ categories may be related to cardiorespiratory fitness. Amireault & Godin [85] reported that healthy adults (n = 100) who score in the “active” category have a higher VO2 max and lower body fat percentage than those who score in the “insufficiently active or sedentary” category.

### Caffeine consumption & withdrawal

The Caffeine Consumption Questionnaire-Revised (CCQ-R; [86]) was completed by participants at their first experimental session and used to estimate the amount of caffeine in milligrams that participants would consume daily. The CCQ-R provides images of caffeine-containing products (i.e., foods, beverages, and drugs) and participants are asked to “indicate how many servings per week [they] normally consume of each item”.

The Caffeine Withdrawal Symptom Questionnaire (CWSQ; [87]) was also completed by participants to assess the presence, type, and severity of caffeine withdrawal symptoms. The CWSQ consists of 23 items which focus on seven symptom clusters: 1) fatigue/drowsiness, 2) low alertness/difficulty concentrating, 3) mood disturbances, 4) low sociability/motivation to work, 5) nausea/upset stomach, 6) flu-like feelings, and 7) headache. Participants report the severity of each symptom using a 5-point, Likert-type scale ranging from 0 (not at all) to 4 (extremely). Potential scores range from 0–92; higher scores indicate a greater number of symptoms and greater symptom severity. The CWSQ was to determine if there was differences in participants’ baseline level of caffeine withdrawal, as this could potentially affect their response to the subsequent treatment**.**

### Psychomotor Vigilance Task (PVT)

Participants completed a 5-minute psychomotor vigilance task (PVT) as an objective assessment of their mental fatigue levels. The PVT was derived from a freely available PC-PVT software (Psychomotor Vigilance Task software for PC, v. 1.1.0), which was downloaded from http://bhsai.org/downloads/pc-pvt/. The PC-PVT software was originally developed and validated by Khitrov and colleagues [88]. The 5-minute PVT protocol we utilized was adapted from Thompson et al. [89]. The test was administered using a PC (Stream 11, HP Inc., Palo Alto, CA) and an optical gaming mouse (Pavilion Gaming Mouse 300, HP Inc., Palo Alto, CA).

RT in milliseconds, false starts, and lapses were recorded for each trial. RT was identified as the primary objective mental fatigue outcome. False starts were defined as responses prior to stimulus presentation or a RT < 100 ms [90]. Basner et al. [91] demonstrated that reducing the threshold for lapses from RT > 500 ms (seen in the traditional 10-minute PVT) to RT > 355 ms supported the convergent validity of a shorter, 3-minute PVT. Accordingly, for our purposes, lapses were defined as RT > 400 ms during the 5-minute PVT.

### Mental and Physical State and Trait Energy and Fatigue Scales (MPSTEFS)

Participants completed the Mental and Physical State and Trait Energy and Fatigue Scales (MPSTEFS) to provide a comprehensive assessment of state and trait fatigue and energy [92]. The 24-item MPSTEFS can be broken down into 8 sub-scales, each represented by 3 items: 1) trait mental fatigue, 2) state mental fatigue, 3) trait mental energy, 4) state mental energy, 5) trait physical fatigue, 6) state physical fatigue, 7) trait physical energy, and 8) state physical energy. On both state and trait scales, energy-related items asked about feelings of energy, vigour, and pep, whereas fatigue-related items asked about feelings of fatigue, exhaustion, and being worn out. For state sub-scale items, participants reported the intensity of their feelings in the current moment using a visual analog scale. Combining the three items on each state subscale created a potential score range from 0 (least intensity) to 300 (greatest intensity). In contrast, for trait sub-scale items, participants indicated the frequency of their feelings using a 5-point, Likert-type scale. Combining the three items on each trait subscale created a potential score range from 0 (lowest frequency) to 12 (highest frequency). The state mental fatigue subscale was identified as the primary subjective mental fatigue outcome.

### Heart rate & blood pressure

HR (bpm) was assessed using a wearable (chest strap) heart rate monitor (Polar H10 Wearlink + Coded Transmitter, Polar Electro Inc., Lake Success, NY, USA). Blood pressure (mmHg) using an electronic sphygmomanometer (Omron BP7455CAN, Omron Healthcare Co., Kyoto, Japan).

Participant study characteristics are summarized in Table 1.

### Procedure

Eligible participants were invited to complete three 90-minute in-lab experimental sessions. All sessions were completed between 8:00 a.m. and 1:00 p.m., each separated by at least 48 hours [93]. Moreover, participants began all three sessions at the same time of day (e.g., 9:30 a.m. Monday, Wednesday, and Friday). Participants were asked to abstain from alcohol and drugs for at least 24 hours prior to their experimental session. They were asked to abstain from caffeine and very intense physical exercise (such as running, weightlifting, circuit training, or sports) at least 12 hours prior to their experimental session. Furthermore, to control the effects of post-prandial somnolence, participants were instructed to refrain from consuming large meals at least 90 minutes prior to their experiment [94,95]. Additionally, participants were asked to adhere to their regular sleep schedule the night prior to their session and record their wake-up time and bedtime.

Upon their initial arrival at the lab, participants were asked to complete the PAR-Q+ and CCQ-R. Next, participants were given the MPSTEFS, GLTEQ, and CWSQ. Baseline HR and BP were assessed following the completion of these questionnaires.

Afterward, participants completed a baseline 5-minute PVT. Then, participants completed one of the three treatments according to their randomization: 1) aerobic exercise condition (EX), 2) caffeine consumption condition (CC), or 3) placebo consumption condition (PC). Participants were randomized to one of six Williams sequences (e.g., ABC, BCA, CAB, ACB, CBA, BAC) using permuted blocks of size 6 to achieve near-equal allocation. The randomization list was generated prior to enrollment using an online randomization tool (random.org), and assignments were concealed in sealed opaque envelopes until allocation. A single researcher (N.F.) conducted the randomization. The researcher administering the treatment (A.S.) and participants were both blinded to PC and CC treatment allocation. Following treatment, participants then completed a 30-minute Stroop task to induce mental fatigue. Finally, post-fatigue induction, participants engaged in a second 5-minute PVT. HR, BP, and subjective state mental fatigue were assessed at baseline, post-treatment (EX, CC, or PC), and post-fatigue induction. Participants were compensated with a $15 Tim Horton’s Gift Card for their participation.

### Statistical analyses

The level of significance was accepted at p < 0.05 for all tests (i.e., alpha level of 0.05). PVT, questionnaire, Stroop task, and physiological data were all assessed via repeated measures ANOVA with a between-subject factor of Treatment (i.e., Exercise, Caffeine, or Placebo) and a within-subject factor of Time. All randomized participants (n = 26) were included in analyses. Prior to ANOVAs, Mauchly’s tests were conducted to assess the assumption of sphericity. Significant results from Mauchly’s test were reported, and degrees of freedom were corrected using Huynh-Feldt estimates of sphericity [96]. When significant effects were identified via repeated measure ANOVA, post-hoc Bonferroni-corrected t-tests were conducted. When conducting post-hoc tests, significant interactions took precedence over significant main effects. Moreover, pairwise comparisons were reported across either treatment or time (e.g., baseline CC compared to baseline PC or post-treatment CC, but not post-treatment PC). Effect sizes (η_p_^2^, Cohen’s d) accompany all reported findings. Data were analyzed using JASP software (Version 0.18.3).

Physiological data and Stroop task data were analyzed as a manipulation check to gauge whether participants had physically and cognitively exerted themselves, respectively. To assess participants’ Stroop task performance over the course of mental fatigue induction, each participant’s Stroop task data was divided into the first 10%, middle 10%, and final 10% of trials completed. Given a participant who completes 1000 trials, the division would be as follows: first 10%, trials 0–100; middle 10%, trials 450–550; and final 10%, trials 900–1000. Stroop task trials were excluded if the trials were identified as outliers. Outliers were identified as being at least ± 3 standard deviations (SDs) from a participant’s condition-specific mean (e.g., a participant’s mean for incongruent trials) [97]. Outlier removal established upper and lower bounds for RT similar to lapses and false starts in the PVT, respectively. The existence of a speed-accuracy trade-off between Stroop task RT and Stroop task accuracy was evaluated via correlational analysis between accuracy and RT at each time point [98,99].

## Results

### Treatment equivalency check

One-way ANOVAs were conducted for CWSQ scores and hours of sleep across treatments. There were no reliable differences between treatments in baseline CWSQ (F(2, 75) = 1.077, p = 0.346, η_p_^2^ = 0.028) and hours of sleep (F(2, 75) = 0.935, p = 0.397, η_p_^2^ = 0.024).

### Manipulation check

#### Stroop task RT (Fatigue induction).

Participants completed an average of 1802 trials ± 295 trials. Approximately 1.8% of these trials were identified as outliers and subsequently removed from analysis.

For control trials (i.e., coloured rectangle, rather than word, stimuli), Mauchly’s test indicated that the assumption of sphericity had been violated, χ^2^ (2) = 9.885, p = 0.007. The main effect of time was not significant, F(1.818, 136.337) = 1.210, p = 0.299, η_p_^2^ = 0.016. Also, the main effect of treatment was not significant, F(2, 75) = 0.205, p = 0.815, η_p_^2^ = 0.005. Moreover, there was no significant interaction between time and treatment F(3.636, 136.337) = 1.790, p = 0.140, η_p_^2^ = 0.046.

For congruent trials, the main effect of time was not significant, F(2, 150) = 0.003, p = 0.997, η_p_^2^ < 0.001. The main effect of treatment was not significant, F(2, 75) = 0.235, p = 0.792, η_p_^2^ = 0.006. Moreover, there was no significant interaction between time and treatment, F(4, 150) = 0.350, p = 0.844, η_p_^2^ = 0.009.

**Fig 3:**
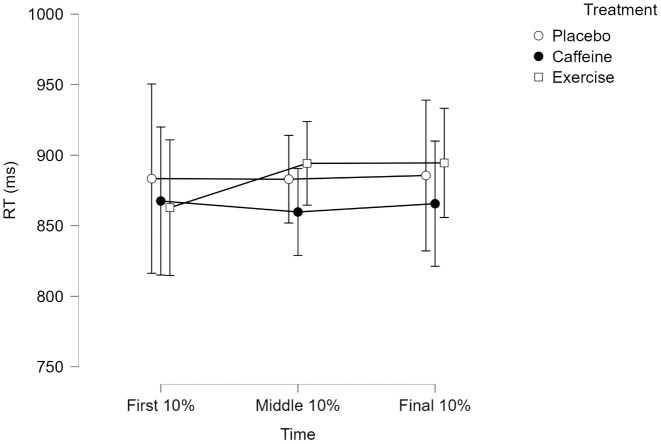
Incongruent Stroop trial RT. Values represent means. Error bars represent 95% CI.

For incongruent trials, Mauchly’s test indicated that the assumption of sphericity had been violated, χ^2^ (2) = 37.156, p < 0.001. The main effect of time was not significant, F(1.454, 109.065) = 0.187, p = 0.759, η_p_^2^ = 0.002. The main effect of treatment was not significant, F(2, 75) = 0.091, p = 0.913, η_p_^2^ = 0.002. Moreover, there was a non-significant interaction between time and treatment, F(2.908, 109.065) = 0.265, p = 0.844, η_p_^2^ = 0.007. Fig 3 depicts incongruent trial RT means and confidence intervals (CIs) across treatments.

### Stroop task accuracy (fatigue induction)

For control trials, the main effect of time was significant, F(2, 150) = 16.749, p < 0.001, η_p_^2^ = 0.183. However, the main effect of treatment was not significant, F(2, 75) = 0.010, p = 0.990, η_p_^2^ < 0.001. Also, there was no significant interaction between time and treatment, F(4, 150) = 0.877, p = 0.479, η_p_^2^ = 0.023. Pairwise comparisons using a Bonferroni correction revealed a significant decrease in accuracy between first and middle (t(25) = 4.301, p < 0.001, d = 0.523), and first and final time points (t(25) = 5.504, p < 0.001, d = 0.669). However, there was no difference between the middle and final time points (t(25) = 1.204, p = 0.692, d = 0.146).

For congruent trials, the main effect of time was significant, F(2, 150) = 13.182, p < 0.001, η_p_^2^ = 0.149. However, the main effect of treatment was not significant, F(2, 75) = 0.064, p = 0.938, η_p_^2^ = 0.002. Also, there was no significant interaction between time and treatment, F(4, 150) = 0.450, p = 0.772, η_p_^2^ = 0.012. Pairwise comparisons using a Bonferroni correction revealed a significant decrease in accuracy between first and middle (t(25) = 4.466, p < 0.001, d = 0.603), and first and final time points (t(25) = 4.428, p < 0.001, d = 0.598). However, there was no difference between the middle and final time points (t(25) = −0.038, p = 1.000, d = −0.005).

**Fig 4:**
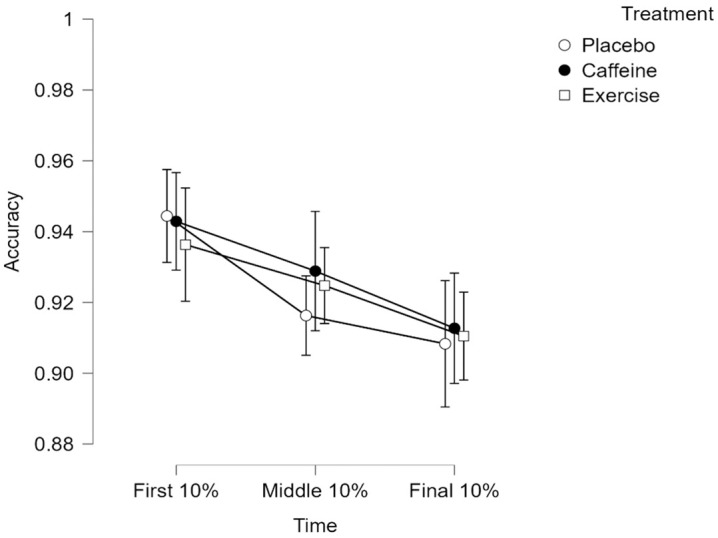
Incongruent Stroop trial accuracy. Values represent means. Error bars represent 95% CI.

For incongruent trials, the main effect of time was significant, F(2, 150) = 14.670, p < 0.001, η_p_^2^ = 0.164. However, the main effect of treatment was not significant, F(2, 75) = 0.095, p = 0.909, η_p_^2^ = 0.003. Also, there was no significant interaction between time and treatment, F(4, 150) = 0.427, p = 0.789, η_p_^2^ = 0.011. Pairwise comparisons using a Bonferroni correction revealed a significant decrease in accuracy between first and middle (t(25) = 3.145, p < 0.006, d = 0.331), first and final (t(25) = 5.392, p < 0.001, d = 0.568). However, there was no reliable difference between the middle and final time points (t(25) = 2.247, p = 0.078, d = 0.237). Correlational analyses between RT and accuracy revealed non-significant interactions at all time points (all p’s > 0.05). Fig 4 depicts incongruent trial accuracy means and CIs across treatments.

### Physiological results

Data on HR, SBP, and DBP can be found in the supporting information (S1 File).

### PVT (objectively measured fatigue)

#### RT (primary outcome).

**Fig 5:**
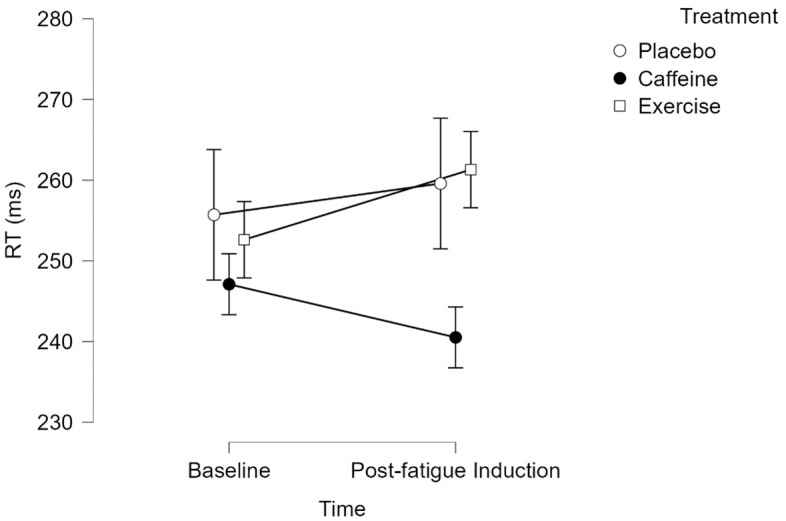
PVT RT. Values represent means. Error bars represent 95% CI.

The main effect of time was not significant, F(1, 75) = 0.744 p = 0.391, η_p_^2^ = 0.010. The main effect of treatment was not significant, F(2, 75) = 2.297, p = 0.108, η_p_^2^ = 0.058. However, there was a significant interaction between time and treatment, F(2, 75) = 3.802, p = 0.027, η_p_^2^ = 0.092. Pairwise comparisons for time and treatment interactions using a Bonferroni correction were non-significant between all treatment time points (all p’s > 0.05). Fig 5 depicts PVT RT means and CIs across treatments.

### Lapses & false starts (Primary Outcome)

Data on PVT Lapses and False Starts can be found in the supporting information (S1 File).

### Self-reported mental and physical state (subjectively measured fatigue)

#### Mental fatigue (primary outcome).

**Fig 6:**
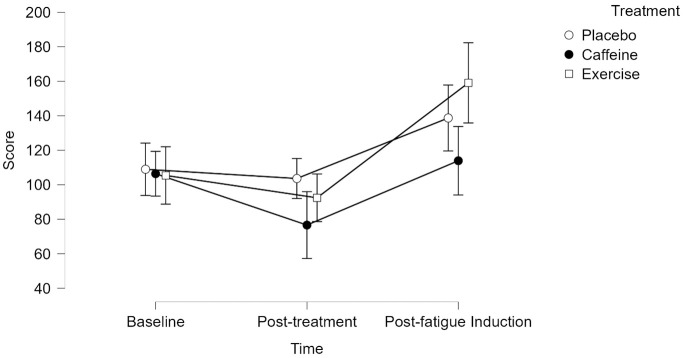
State Mental Fatigue Scores. Values represent means. Error bars represent 95% CI.

Mauchly’s test indicated that the assumption of sphericity had been violated, χ^2^ (2) = 17.403, p < 0.001. The main effect of time was significant, F(1.686, 126.453) = 23.723, p < 0.001, η_p_^2^ = 0.240. The main effect of treatment was not significant, F(2, 75) = 1.148, p = 0.323, η_p_^2^ = 0.030. Moreover, there was a non-significant interaction between time and treatment, F(3.372, 126.453) = 2.378, p = 0.066, η_p_^2^ = 0.060. Pairwise comparisons by time using a Bonferroni correction revealed an increase in scores from post-treatment to post-fatigue induction t(25) = −6.781, p < 0.001, d = −0.735) and from baseline to post-fatigue induction (t(25) = −4.437, p < 0.001, d = −0.481). However, there was no significant difference between baseline and post-treatment t(25) = 2.345, p = 0.061, d = 0.254). Fig 6 depicts state mental fatigue means and CIs across treatments.

**Fig 7:**
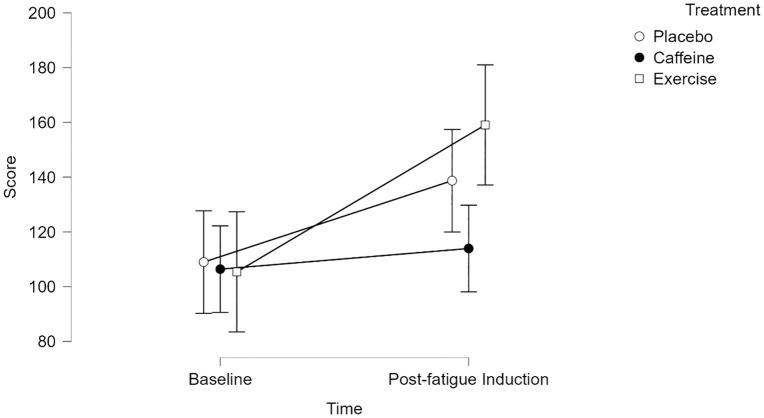
State Mental Fatigue Scores–Post-treatment Data Removed. Values represent means. Error bars represent 95% CI.

To facilitate comparison with the objective primary outcome, state mental fatigue was also analyzed without the post-treatment time point. The main effect of time was significant, F(1, 75) = 16.196, p < 0.001, η_p_^2^ = 0.178. The main effect of treatment was not significant, F(2, 75) = 1.095, p = 0.340, η_p_^2^ = 0.028. Moreover, there was a significant interaction between time and treatment, F(2, 75) = 3.129, p = 0.050, η_p_^2^ = 0.077. Pairwise comparisons using a Bonferroni correction revealed a significant increase in mental fatigue from baseline EX to post-fatigue induction EX (t(25) = −4.115, p = 0.001, d = −0.843). Fig 7 depicts state mental fatigue means and CIs across treatments without post-treatment data.

### Other fatigue & energy outcomes

Data on mental energy, physical fatigue, and physical energy can be found in the supporting information (S1 File).

## Discussion

The present investigation sought to compare the effects of three treatments on subjective and objective assessments of mental fatigue when administered prior to mental fatigue induction: 1) a 20-minute, moderate-intensity bout of cycling; 2) consumption of 2.5 mg/kg caffeine; and 3) placebo. To begin, we will discuss the Stroop task fatigue induction, followed by HR and BP, and then the objective and subjective fatigue outcomes.

### Mental fatigue induction (Stroop task)

The results indicated that participants’ RTs did not reliably lengthen as a function of time in the Stroop task. Since RT was resilient to “Time on Task” effects, a decrement in accuracy over time can be anticipated [98].

Accuracy significantly decreased over time across all three Stroop trial conditions, irrespective of treatment. This suggests that participants were indeed cognitively exerting themselves during the Stroop task [77]. Although these results suggest successful mental fatigue induction, they should be interpreted with caution. Mangin et al. [100] found declines in performance during a 30-minute Stroop task were correlated with subjective measures of boredom (r = −0.332, p = 0.003). Several past studies have suggested that when in a mentally fatigued state, cognitive compensatory mechanisms reallocate attentional resources to preserve either RT or accuracy; hence, a speed-accuracy trade-off is commonly observed in incongruent Stroop trials [98,100–102]. However, our correlational analyses did not demonstrate a significant speed-accuracy trade-off.

In summary, Stroop task results reflect that participants exerted cognitive effort throughout the 30-minute induction. The primary outcomes must be reviewed to determine whether mental fatigue was effectively induced.

### Physiological measures

Full discussion of physiological measures can be found in the supporting information (S1 File). In summary, physiological variables increased as anticipated in the exercise and caffeine consumption conditions and remained relatively stable in the placebo consumption condition.

### PVT RT (primary objective outcome)

PVT RT results indicated a significant time-by-treatment interaction effect with a medium effect size (η_p_^2^ = 0.092; [103]). Thus, a 30-minute Stroop task did result in a significant difference in objective mental fatigue measured via 5-minute PVT performance. However, no significant differences were found in post-hoc Bonferroni-corrected pairwise comparisons. This contradicts the hypothesis that the Stroop task would result in differences in PVT RT between treatments from baseline to post-fatigue induction. Furthermore, this is in line with the findings of Jeyarajan et al. [55] which suggest that a 20-minute bout of moderate-intensity exercise imparts no reliable benefit to mental fatigue resilience measured via PVT Differences in PVT duration may partially account for the divergence in PVT findings. Roach et al.’s [104] comparison of 3-minute, 5-minute, and 10-minute PVT formats suggested that the 5-minute format was more sensitive to the effects of fatigue.

### State mental fatigue (primary subjective outcome)

As anticipated, participants reported significantly greater subjective mental fatigue from post-treatment to post-fatigue induction (d = −0.735, medium effect size; [105]). This suggests that the 30-minute Stroop task successfully induced subjective mental fatigue. Mechanistically, this may be due to the accrual of neurometabolites (e.g., adenosine, glutamate) during task completion, leading to greater feelings of effort [106,107]. Placebo consumption, caffeine consumption, and exercise treatments did not demonstrate a differential impact on mental fatigue status (i.e., no significant time by treatment interaction), contradicting our hypothesis. However, it should be acknowledged that after removing the post-treatment time point, the time by treatment interaction effect was significant (η_p_^2^ = 0.077, medium effect size; [103]), and pairwise comparisons using a Bonferroni correction revealed a significant increase in mental fatigue from baseline exercise to post-fatigue induction exercise (d = −0.843, large effect size, [105]). Thus, exercise may have failed to mitigate subjective mental fatigue.

### Comparing objective and subjective primary outcomes

In both subjective and objective primary outcomes, exercise did not appear to mitigate mental fatigue. The effect of exercise on cognition is modulated by exercise intensity, duration, and participants’ fitness [35]. The present investigation chose a 20-minute bout of moderate-intensity exercise since this has been shown to robustly improve cognitive function (esp. executive function). However, this may not necessarily reflect the optimal dosage of exercise to support mental fatigue resilience. Participants’ fitness status may also exert a differential impact on exercise’s relationship with mental fatigue than on cognitive function. Furthermore, the present study is unable to conclude if the exercise treatment achieved relevant changes in CBF or neurometabolite concentrations, as these variables were not assessed. Differences in participants’ mental fatigue were found in our subjective outcome, but not in our objective outcome. Subjective feelings of mental fatigue have previously been suggested to precede objective performance decrements [108–110]. Among the earliest to suggest this temporal lag was Thorndike [111]. In his seminal mental fatigue investigation, he noted, “We can feel mentally fatigued without being so, so that the feelings serve as a sign to stop working long before our actual ability to work has suffered any important decrease”. Accordingly, a longer duration of mental fatigue induction may be required to elicit objectively measurable mental fatigue than subjectively measurable mental fatigue. This may, in part, explain the differences in the present investigations’ subjective and objective outcomes.

In both subjective and objective outcomes, caffeine and placebo did not exhibit a reliable difference in pairwise comparisons. This may be due to several factors. It may be because both treatments are mitigating mental fatigue to a similar degree (i.e., placebo effect). Conversely, caffeine has a distinct bitter flavour which may not have been replicated adequately by corn starch. This may have contributed to unblinding between the conditions. The dosage of caffeine may have been inappropriate to produce a significant difference in mental fatigue resilience. Alternatively, the severity of the mental fatigue induction may have been insufficient to produce reliable post-hoc differences from the caffeine and placebo treatments.

### Other fatigue and energy outcomes

As anticipated, participants reported significantly less subjective mental energy from post-treatment to post-fatigue induction, and baseline to post-fatigue induction. This further supports participants having mentally exerted themselves during the 30-minute Stroop task. Loy et al.’s [112] meta-analysis reported that a 21–40-minute bout of light-to-moderate intensity exercise decreased self-reported fatigue, only when energy levels were concurrently increased. This may also explain, in part, why the exercise treatment was ineffective: feelings of mental energy were not significantly increased, an effect which Loy et al. [112] suggest may be modulated by the intensity and duration of the exercise bout.

Participants reported significantly greater physical fatigue and lesser mental energy from baseline to post-fatigue induction, and post-treatment to post-fatigue induction. These findings supplement an existing body of evidence that suggests mental and physical fatigue may share a common process (i.e., central fatigue) [53,113–117].

In summary, mental energy and physical fatigue & energy scores are in agreement with state mental fatigue and physiological data. These results altogether support that the 30-minute Stroop task induced subjective mental fatigue, and the 20-minute moderate-intensity cycling treatment posed the anticipated physical demands.

### Limitations, implications, future directions

The present investigation had several limitations. In recognition of the transience of post-exercise effects, 5-minute PVT performance was not measured post-treatment (i.e., participants progressed to the fatigue induction immediately following the MPSTEFS). Future investigations should aim to objectively assess mental fatigue status both immediately post-treatment and following mental fatigue induction. The 3-minute PVT may be an effective tool for these purposes, minimizing the time lag between post-treatment and fatigue induction. There were no EEG or HRV measures included in the present study. Future research should utilize these tools to gather insight into participants’ mental fatigue status during fatigue induction and PVT assessment. Also, a 30-minute Stroop task was utilized for fatigue induction. Future studies should compare various tasks and durations to find the optimal way to induce mental fatigue. Further, recent work by Hassan et al. [118] demonstrated that employing a cognitive task battery may be a promising method to induce mental fatigue. As a pragmatic control measure, we advised participants to avoid “large meals” within 90 minutes of testing; however, future work should impose stricter nutritional control to reduce the influence of post-prandial states. Moreover, powdered caffeine may have had a bitter taste which influenced participants’ reactions to their treatment. Future studies should attempt to find an alternative taste-matched placebo treatment to reduce any expectancy effects. Furthermore, the present study specifically examined the efficacy of a 20-minute bout of moderate-intensity aerobic exercise. Future investigations should explore various durations and intensities of exercise to find the optimal dose to mitigate mental fatigue. Participants’ self-reported behaviour was relied upon for caffeine abstinence and hours of sleep. Future investigations may benefit from confirming caffeine abstinence empirically and obtaining a more thorough assessment of sleep quality and volume; this may be achieved by collecting and processing a saliva sample from participants upon lab arrival and having participants complete a validated sleep assessment, such as the Pittsburgh Sleep Quality Index [119], respectively. Participants were not compared based on their daily caffeine consumption. Future studies should compare the effects of caffeine on mental fatigue in those with varying degrees of daily caffeine consumption, including those who are caffeine naïve. The participants in the present study were primarily young, active, and highly educated. Future research should examine the effects of caffeine and acute exercise on mental fatigue across various age groups, physical activity levels, and education levels. Lastly, the present study evaluated the individual effects of aerobic exercise and caffeine consumption on mental fatigue; future studies should investigate the effects of combining these interventions on mental fatigue.

At present, it appears that a 20-minute bout of moderate-intensity exercise, completed before mental fatigue induction, does not aid in mitigating mental fatigue accrual. Further investigations are warranted to explore the potential of varying exercise intensities and durations in mitigating mental fatigue and identify non-harmful alternatives to caffeine consumption. Creatine monohydrate supplementation has shown promise in mitigating mental fatigue [120]. The effects of combining interventions such as creatine monohydrate supplementation and acute exercise should be evaluated in future research. Such investigations not only serve to elucidate the efficacy of aerobic exercise and other caffeine alternatives in mitigating mental fatigue but ultimately provide guidance for health-related interventions for the general population.

## Conclusion

Findings from the present study suggest that a 30-minute Stroop task is effective in inducing subjective mental fatigue, but not objective mental fatigue. Consumption of 2.5 mg/kg of caffeine may mitigate subjective mental fatigue similar to placebo consumption in adult caffeine consumers. Furthermore, a 20-minute bout of moderate-intensity aerobic exercise may not be an effective method of mitigating fatigue. Further investigations are warranted to explore the potential of varying exercise intensities and durations in mitigating mental fatigue. Moreover, future research into the mechanisms underlying mental fatigue may aid in the development of more efficacious mental fatigue interventions.

## Supporting information

S1 File Supplementary Data & Results. (DOCX)

S2 File CONSORT Checklist. (DOCX)
