# Supplementary material for: Differential effects of caffeine, acute aerobic exercise, and placebo on mental fatigue
Source: PLoS One. 2026 Apr 30;21(4):e0348279. doi: 10.1371/journal.pone.0348279 (PMC13132226; doi:10.1371/journal.pone.0348279)
Supplement: S1 File — (DOCX) [file pone.0348279.s001.docx]

**S1 Text: Additional Physiological, PVT, Fatigue & Energy Information**

**Physiological Measures**

***HR***

The main effect of time was significant, F(2, 150) = 41.611, p < 0.001, η_p_^2^ = 0.357. The main effect of treatment was significant, F(2, 75) = 6.036, p = 0.004, η_p_^2^ = 0.139. Also, there was a significant interaction between time and treatment, F(4, 150) = 47.718, p < 0.001, η_p_^2^ = 0.560. In terms of EX, HR increased from baseline to post-treatment (t(25) = −15.040, p < 0.001, d = −1.561), then decreased to post-fatigue induction (t(25) = 12.199, p < 0.001, d = 1.266). Post-treatment EX HR was greater than PC (t(25) = 6.362, p < 0.001, d = 1.765) and CC (t(25) = 7.140, p < 0.001, d = 1.980). CC demonstrated a decrease in HR between baseline and post-fatigue induction (t(25) = 3.438, p = 0.027, d = 0.357). Figure A depicts HR means and CIs across treatments.


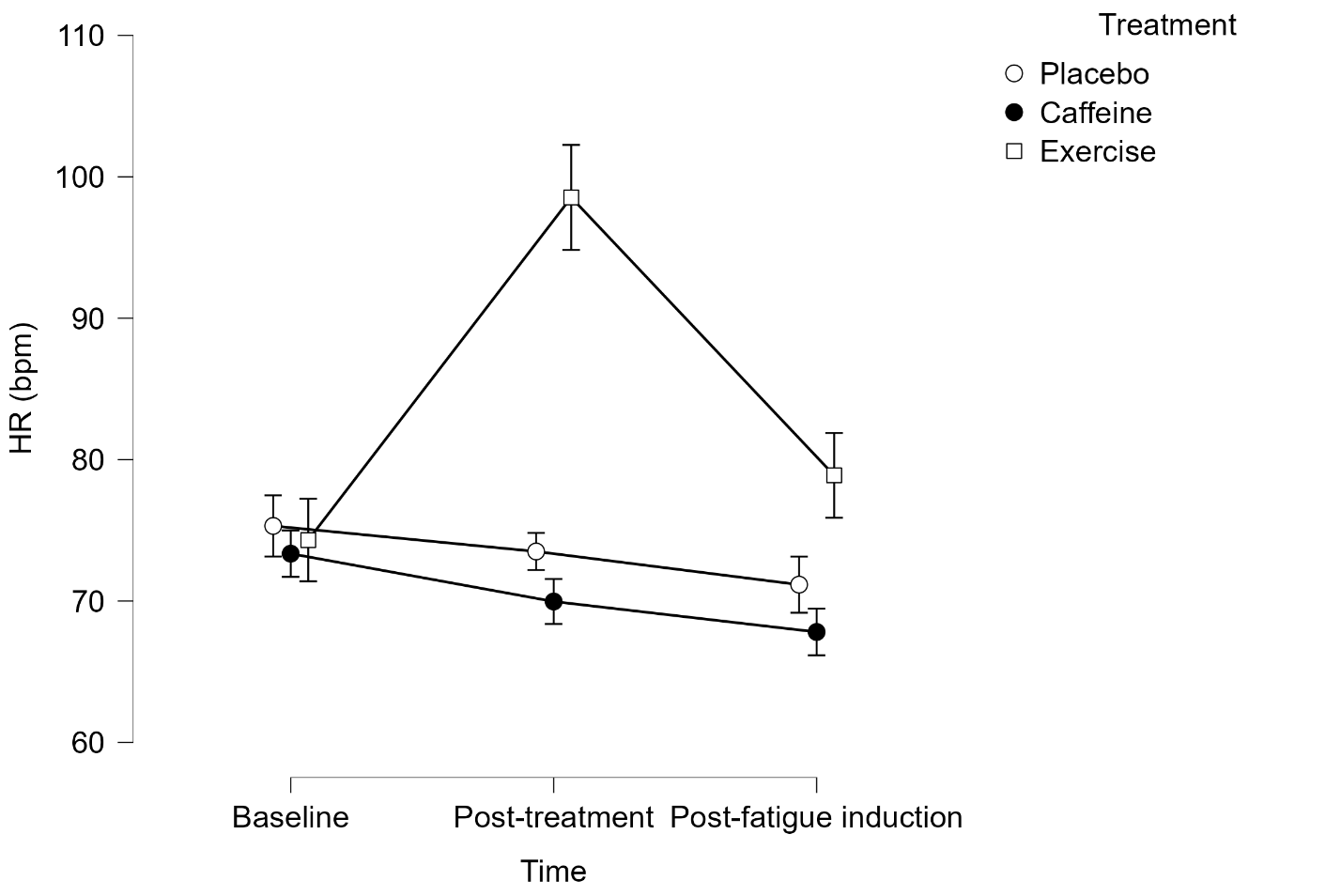


**Fig A**. **HR**. Values represent means. Errors bars represent 95% CI.

***SBP***

The main effect of time was significant F(2, 150) = 8.571, p < 0.001, η_p_^2^ = 0.103. The main effect of treatment was not significant, F(2, 75) = 0.027, p = 0.973, η_p_^2^ < 0.001. However, there was a significant interaction between time and treatment, F(4, 150) = 8.409, p < 0.001, η_p_^2^ = 0.183. In terms of EX, SBP decreased from post-treatment to post-fatigue induction (t(25) = 6.198, p < 0.001, d = 0.868) and from baseline to post-fatigue induction (t(25) = 3.510, p = 0.021, d = 0.492). Figure B depicts SBP means and CIs across treatments.


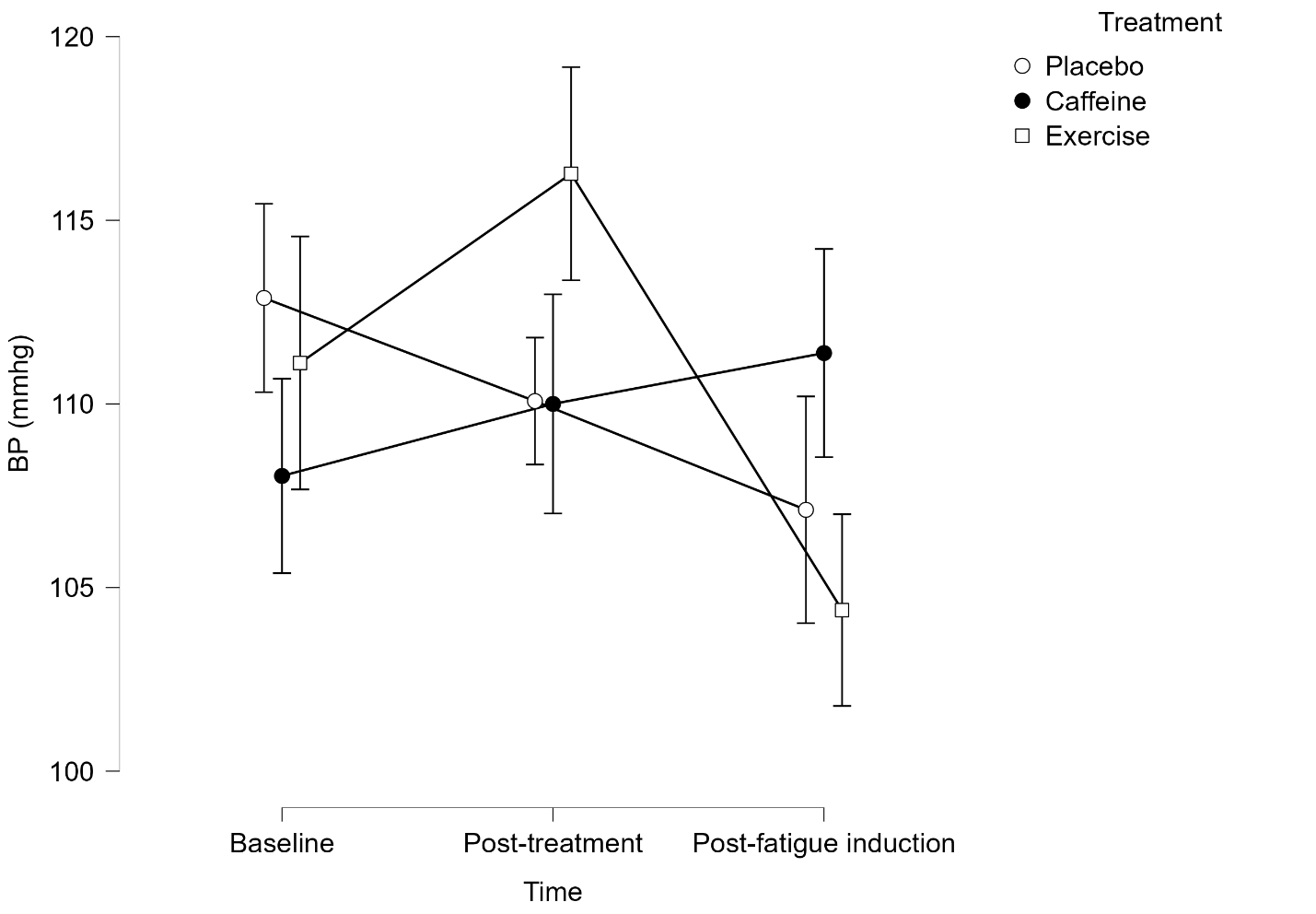


**Fig B.** **SBP**. Values represent means. Errors bars represent 95% CI.

***DBP***

The main effect of time was significant F(2, 150) = 9.404, p < 0.001, η_p_^2^ = 0.111. The main effect of treatment was not significant, F(2, 75) = 0.059, p = 0.945, η_p_^2^ = 0.002. However, there was a significant interaction between time and treatment, F(4, 150) = 5.455, p < 0.001, η_p_^2^ = 0.127. Pairwise comparisons using a Bonferroni correction revealed that DBP significantly increased between baseline EX and post-treatment EX (t(25) = −4.685, p < 0.001, d = −0.730) and between baseline post-treatment EX and post-fatigue induction EX (t(25) = 3.707, p = 0.011, d = 0.577). Also, DBP increased between baseline CC and post-fatigue induction CC (t(25) = −3.758, p = 0.009, d = −0.585). Figure C depicts DBP means and CIs across treatments.


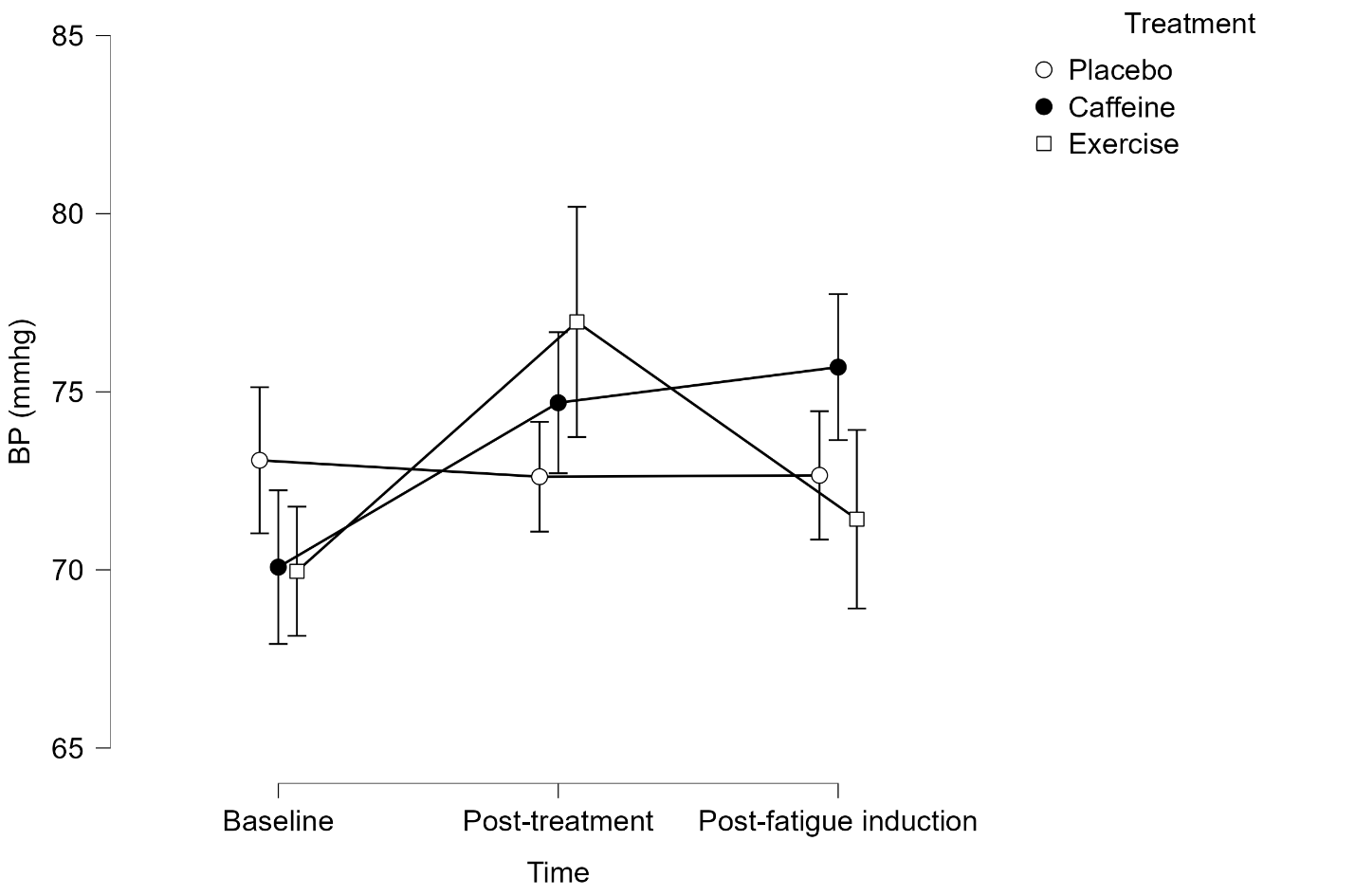


**Fig C. DBP**. Values represent means. Errors bars represent 95% CI.

**PVT**

***Lapses***

The main effect of time was not significant, F(1, 75) = 3.398 p = 0.069, η_p_^2^  = 0.043. The main effect of treatment was not significant, F(2, 75) = 1.835, p = 0.167, η_p_^2^ = 0.047. Moreover, there was non-significant interaction between time and treatment, F(2, 75) = 1.888, p = 0.158, η_p_^2^ = 0.048. Figure D depicts PVT lapse means and CIs across treatments.


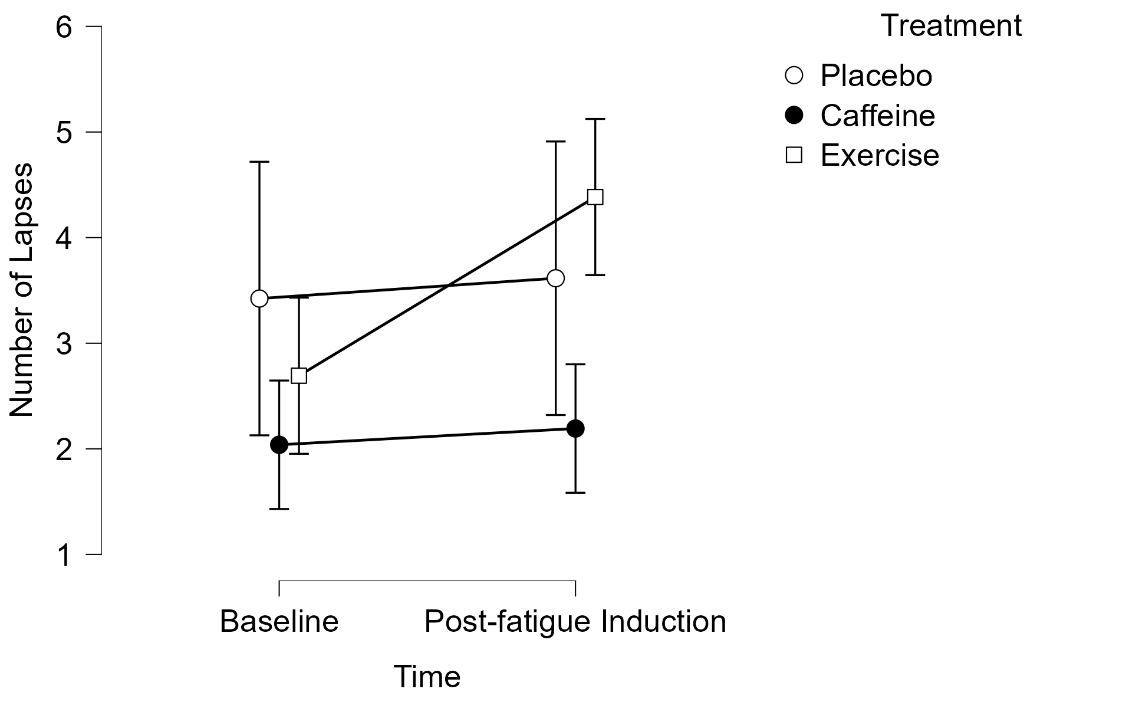


Fig D. PVT Lapses by Time and Treatment. Values represent means. Errors bars represent 95% CI.

***False Starts***

Participants averaged 0.269 ± 0.617 false starts at baseline and 0.346 ± 0.599 post-fatigue induction. RM ANOVAs were not conducted since mean false starts were < 1 at both timepoints.

**Self-reported Mental and Physical State**

***Mental Energy***

Mauchly’s test indicated that the assumption of sphericity had been violated, χ^2^ (2) = 17.801, p < 0.001. The main effect of time was significant, F(1.680, 125.994) = 19.781, p < 0.001, η_p_^2^ = 0.209. The main effect of treatment was not significant, F(2, 75) = 0.209, p = 0.812, η_p_^2^ = 0.006. Moreover, there was a non-significant interaction between time and treatment, F(3.360, 125.994) = 1.998, p = 0.110, η_p_^2^ = 0.051. Pairwise comparisons by time using a Bonferroni correction revealed a significant increase in mental energy state scores between post-treatment and post-fatigue induction t(25) = 6.222, p < 0.001, d = 0.800) and baseline and post-fatigue induction t(25) = 3.907, p < 0.001, d = 0.502). However, there was no significant difference between baseline and post-treatment (t(25) = −2.315, p = 0.066, d = −0.297). Figure E depicts state mental energy means and CIs across treatments.


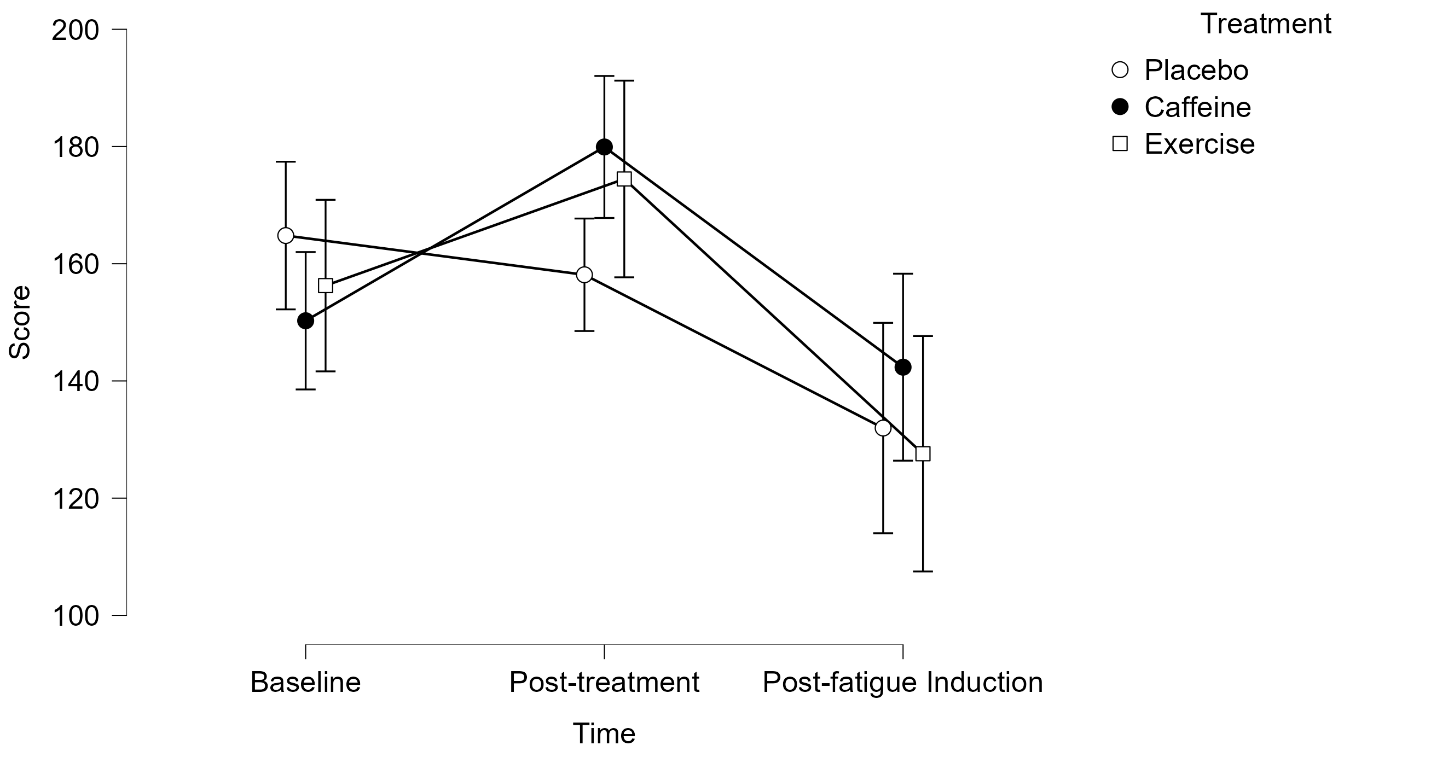


Fig E. State Mental Energy Scores. Values represent means. Errors bars represent 95% CI.

***Physical Fatigue***

The main effect of time was significant, F(2, 150) = 10.636, p < 0.001, η_p_^2^ = 0.124. The main effect of treatment was not significant, F(2, 75) = 0.511, p = 0.728, η_p_^2^ = 0.013. Moreover, there was non-significant interaction between time and treatment, F(4, 150) = 1.025, p = 0.354, η_p_^2^ = 0.027. Pairwise comparisons by time using a Bonferroni correction revealed a significant increase in physical fatigue state scores between post-treatment and post-fatigue induction (t(25) = −4.286, p < 0.001, d = −0.403), and baseline and post-fatigue induction t(25) = −3.618, p = 0.001, d = −0.403). However, there was no significant differences between baseline and post-treatment (t(25) = 0.668, p = 1.000, d = 0.063). Figure F depicts state physical fatigue means
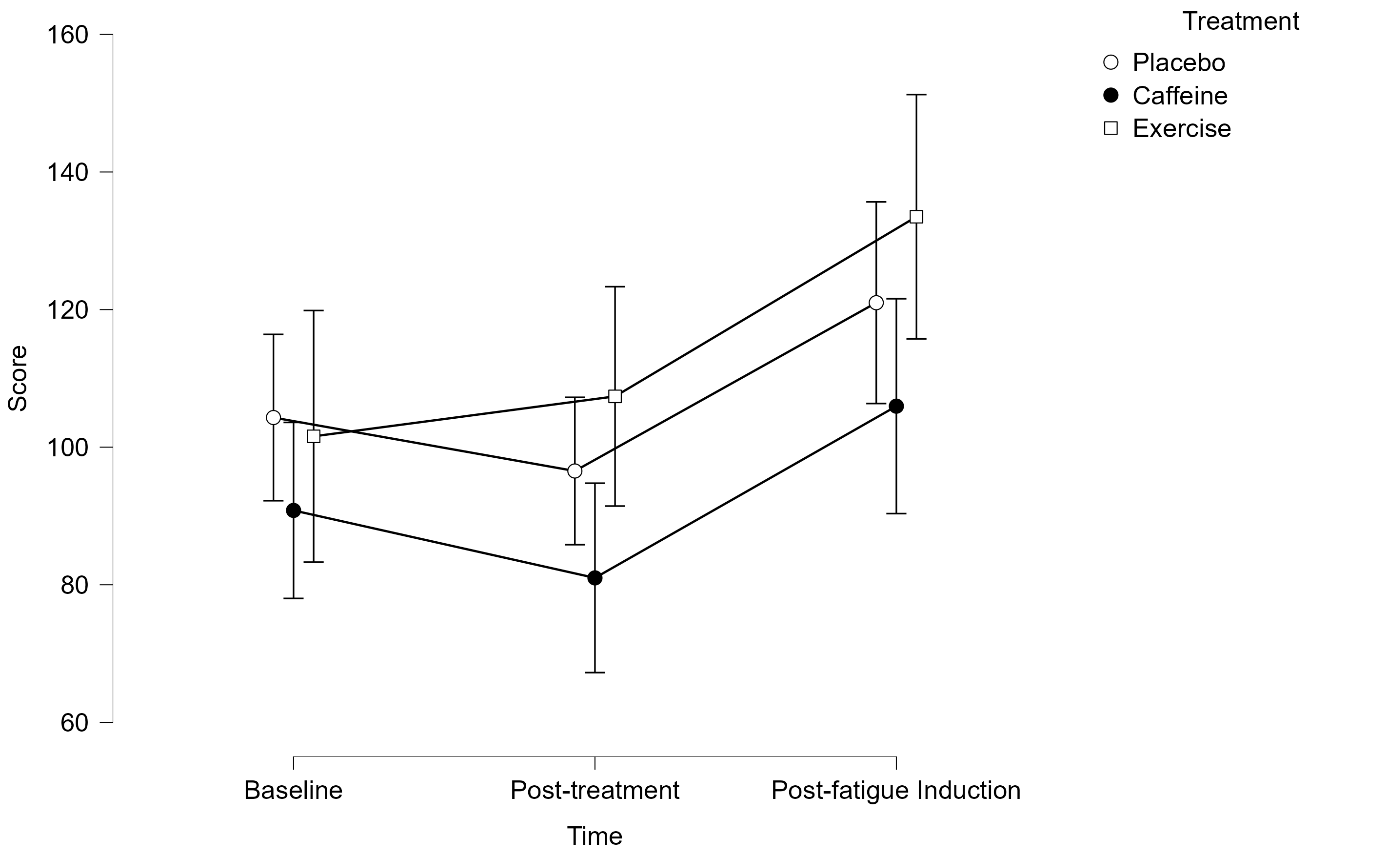
and Cis across treatments.

Fig F. State Physical Fatigue Scores. Values represent means. Errors bars represent 95% CI.

***Physical Energy***

Mauchly’s test indicated that the assumption of sphericity had been violated, χ^2^ (2) = 7.148, p = 0.028. The main effect of time was significant, F(1.875, 140.618) = 15.086, p < 0.001, η_p_^2^ = 0.167. The main effect of treatment was not significant, F(2, 75) = 0.249, p = 0.780, η_p_^2^ = 0.007. Moreover, there was non-significant interaction between time and treatment, F(3.750, 140.618) = 1.075, p = 0.372, η_p_^2^ = 0.028. Pairwise comparisons by time using a Bonferroni correction revealed a significant decrease in physical energy state scores between post-treatment and post-fatigue induction (t(25) = 5.447, p < 0.001, d = 0.544) and between baseline and post-fatigue induction (t(25) = 3.338, p = 0.003, d = 0.334). However, there was no significant differences between baseline and post-treatment (t(25) = −2.109, p = 0.110, d = *
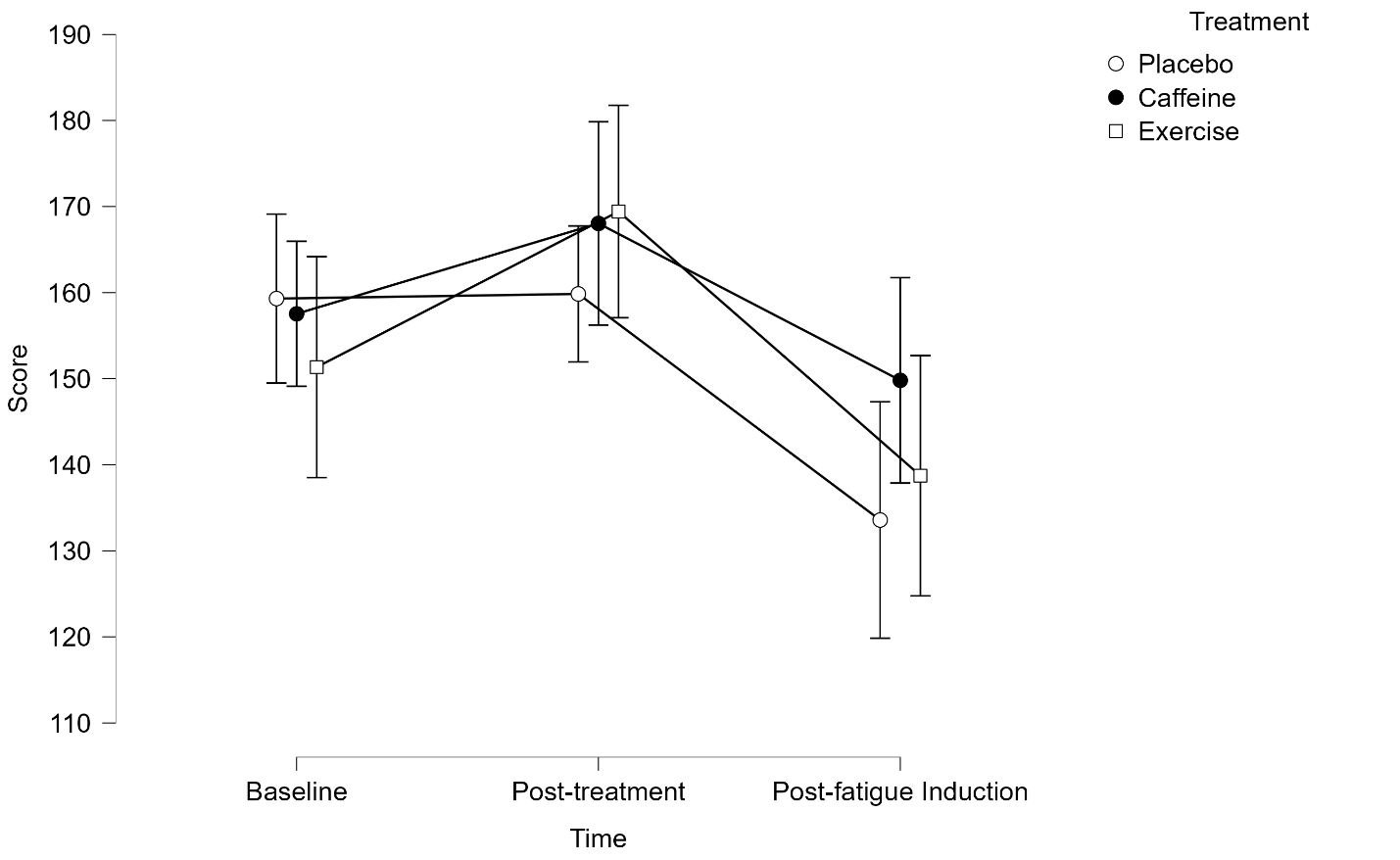
*−0.211). Figure G depicts state physical energy means and CIs across treatments.

Fig G. State Physical Energy Scores. Values represent means. Errors bars represent 95% CI.

***EX***

As anticipated, a 20-minute bout of moderate-intensity cycling resulted in a significant increase in HR and DBP from baseline. There was no significant difference between baseline and post-treatment SBP, however, a positive trend is observable in Fig B (S1 Text). HR remained above baseline levels at the post-fatigue induction time point. In contrast, DBP was not different from baseline and SBP significantly decreased post-fatigue induction (i.e., “post-exercise hypotension”; [1]). HR was significantly greater post-treatment in EX than PC and CC. Post-treatment SBP and DBP were not significantly different between conditions, however a trend, similar to HR, is observable in Figs B and C (S1 Text), respectively. The aforementioned fluctuations in HR and BP are broadly attributable to heightened sympathetic activity to meet energy demands during exercise [2,3].

***CC***

As anticipated, CC resulted in a significant increase in DBP from baseline to post-fatigue induction. Further, a non-significant, positive trend is observable in Fig B (S1 Text) for SBP between baseline and post-fatigue induction. The pressor effects of caffeine have been well-documented by previous literature [4–6]. The relatively small differences between baseline and post-treatment BP suggest that caffeine had not reached peak plasma levels prior to Stroop task fatigue induction; thus, it was likely during Stroop Task completion that caffeine exerted its maximal effects [7]. Moreover, CC resulted in a decrease in HR between baseline and post-fatigue induction. Past literature has suggested that caffeine’s pressor effects are accompanied by small decreases [6,8,9], or no change [10–12], in HR. A decrease in HR may have been observed as a result of “a reflectory bradycardic response to pressor action” (i.e., arterial baroreflex) [7].

***PC***

Participants’ HR and BP remained relatively stable over time in the PC condition, albeit there was a negative trend observable in SBP in Fig B (S1 Text). This stability in HR & BP supports work by Nozaki et al. [13] which reported no significant changes in HR, SBP, or DBP after participants completed four hours of mental fatigue induction (three different cognitive tasks, repeated). Moreover, Ahsberg et al. [14] also found no significant differences in HR or BP following a 60-minute vigilance task; however, there was a significant negative correlation between subjective ratings of fatigue (measured on a 10-point scale) and DBP.

**References**

1. MacDonald JR, MacDougall JD, Hogben CD. The effects of exercise intensity on post exercise hypotension. J Hum Hypertens. 1999;13: 527–531. doi:10.1038/sj.jhh.1000866

2. Maresh CM, Abraham A, De Souza MJ, Deschenes MR, Kraemer WJ, Armstrong LE, et al. Oxygen consumption following exercise of moderate intensity and duration. Europ J Appl Physiol. 1992;65: 421–426. doi:10.1007/BF00243508

3. Romero SA, Minson CT, Halliwill JR. The cardiovascular system after exercise. J Appl Physiol (1985). 2017;122: 925–932. doi:10.1152/japplphysiol.00802.2016

4. Haigh R, Harper G, Fotherby M, Hurd J, Macdonald I, Potter J. Duration of caffeine abstention influences the acute blood pressure responses to caffeine in elderly normotensives. European journal of clinical pharmacology. 1993;44: 549–553.

5. Robertson D, Frölich JC, Carr RK, Watson JT, Hollifield JW, Shand DG, et al. Effects of caffeine on plasma renin activity, catecholamines and blood pressure. New England Journal of Medicine. 1978;298: 181–186.

6. Sung BH, Whitsett TL, Lovallo WR, al’Absi M, Pincomb GA, Wilson MF. Prolonged increase in blood pressure by a single oral dose of caffeine in mildly hypertensive men. American journal of hypertension. 1994;7: 755–758.

7. Nurminen M-L, Niittynen L, Korpela R, Vapaatalo H. Coffee, caffeine and blood pressure: a critical review. Eur J Clin Nutr. 1999;53: 831–839. doi:10.1038/sj.ejcn.1600899

8. Izzo JL, Ghosal A, Kwong T, Freeman RB, Jaenike JR. Age and prior caffeine use alter the cardiovascular and adrenomedullary responses to oral caffeine. The American journal of cardiology. 1983;52: 769–773.

9. Smits P, Pieters G, Thien T. The role of epinephrine in the circulatory effects of coffee. Clinical Pharmacology & Therapeutics. 1986;40: 431–437.

10. Ammon H, Bieck P, Mandalaz D, Verspohl E. Adaptation of blood pressure to continuous heavy coffee drinking in young volunteers. A double-blind crossover study. British journal of clinical pharmacology. 1983;15: 701–706.

11. Casiglia E, Paleari C, Petucco S, Bongiovi S, Colangeli G, Baccilieri M, et al. Haemodynamic effects of coffee and purified caffeine in normal volunteers: a placebo-controlled clinical study. Journal of Human Hypertension. 1992;6: 95–99.

12. Robertson D, Wade D, Workman R, Woosley RL, Oates J, others. Tolerance to the humoral and hemodynamic effects of caffeine in man. The Journal of clinical investigation. 1981;67: 1111–1117.

13. Nozaki S, Tanaka M, Mizuno K, Ataka S, Mizuma H, Tahara T, et al. Mental and physical fatigue-related biochemical alterations. Nutrition. 2009;25: 51–57. doi:10.1016/j.nut.2008.07.010

14. Ahsberg E, Gamberale F, Gustafsson K. Perceived fatigue after mental work: an experimental evaluation of a fatigue inventory. Ergonomics. 2000;43: 252–268. doi:10.1080/001401300184594
